# Effect of tomato variety, cultivation, climate and processing on Sola l 4, an allergen from *Solanum lycopersicum*

**DOI:** 10.1371/journal.pone.0197971

**Published:** 2018-06-14

**Authors:** Elisabeth Kurze, Roberto Lo Scalzo, Gabriele Campanelli, Wilfried Schwab

**Affiliations:** 1 Biotechnology of Natural Products, Technische Universität München, Freising, Germany; 2 Consiglio per la ricerca in agricoltura e l’analisi dell’economia agraria, Unità di ricerca per i processi dell’industria agroalimentare (CREA-IT), Milan, Italy; Chang Gung University, TAIWAN

## Abstract

Tomatoes (*Solanum lycopersicum*) are one of the most consumed vegetables worldwide. However, tomato allergies in patients suffering from birch pollen allergy occur frequently. Due to highly similar protein structures of the tomato allergen Sola l 4 and the major birch pollen allergen Bet v 1, patients cross-react with allergenic proteins from tomato as well as other fruits or vegetables. The aim of this study was to quantify Sola l 4 in various tomatoes differing in color, size and shape for identification of varieties with a reduced allergen level. Therefore, an indirect competitive ELISA using a specific polyclonal Sola l 4 antibody was developed. In addition, two varieties, both cultivated either conventionally or organically and furthermore dried with different methods, were analyzed to investigate the influence of the cultivation method and processing techniques on Sola l 4 level. Within 23 varieties, Sola l 4 content varied significantly between 0.24 and 1.71 μg Sola l 4/g FW. The tomato cultivars Rugantino and Rhianna showed the significantly lowest level, whereas in cultivars Farbini and Bambello the significantly highest concentration was determined. Drying of tomatoes in the oven and by sun resulted in a significant decrease. The thermal instability was verified for the recombinant Sola l 4 emphasizing the results for the native protein in dried tomato samples. Overall, the Sola l 4 content is cultivar-dependent and no correlation between color and Sola l 4 amount was found. During the drying process of tomatoes Sola l 4 level was significantly reduced due to thermal instability. Growing conditions have a minor effect whereas seasonal effects show a more pronounced impact. These findings could extend the knowledge about the allergen level of different tomato varieties and may help to improve food safety to potentially increase the life quality of patients suffering from birch pollen allergy.

## Introduction

Tomato (*Solanum lycopersicum*) is the most commonly grown and consumed vegetable worldwide [1]. Due to its high content of lycopene and ß-carotene, acting as antioxidants and free radical scavenger, tomato is beneficial to health decreasing the risk of cancer and cardiovascular diseases [2]. On the other hand, the consumption of tomatoes can provoke allergic reactions attributed to the presence of various allergenic proteins [3]. The prevalence of food allergy increased during the last decades affecting 3–4% of the adult population and 5% of children [4]. Due to varying geographical distribution of specific pollen allergens as well as local dietary habits, geographical diversities in sensitizations patterns between patients suffering from food allergies occur [5].

A wide range of allergens from plant origin belongs to the pathogenesis-related proteins comprising 17 different protein families [6]. Induced in plants by various stress conditions these proteins are part of the defense response system. Pathogens such as viruses, bacteria or fungi, application of harsh chemicals (herbicides, fungicides), wounding or diverse environmental changes (dryness, UV light) evoke the expression of PR-genes and the synthesis of PR-proteins. The widespread occurrence and the conservation of the PR-10 protein family within the plant kingdom emphasize an important role of this family [7]. The major birch pollen allergen Bet v 1 as well as homologous plant food allergens from *Rosaceae* such as apple (Mal d 1), peach (Pru p 1), cherry (Pru av 1) or strawberry (Fra a 1) belong to the group of intracellular PR-10 proteins with a molecular weight of 16–18 kDa [8]. The presence of a hydrophobic cavity indicates a potential role in binding nonpolar molecules. Many PR-10 proteins share about 50% of amino acid sequence identity [9]. However, cross-reactivity occurs due to the high three-dimensional structure similarity. IgE antibodies recognize similar cross-reactive conformational allergen epitopes of different plant sources [6].

Tomatoes are common sources of plant food allergens [10]. Approximately 1.5% of the population in Northern Europe [11] and up to 16% in Italy [12] is affected by allergy towards tomato. Symptoms of an immunological reaction to tomato can affect the skin (urticarial or dermatitis) but can also lead to oral allergy syndrome, rhinitis or abdominal pain [13]. Food allergies are associated with a reduced life quality and excluding specific fruits or vegetables from the daily diet. People with food allergies against PR-10 homologous allergens develop symptoms after consumption of fresh fruits. On the contrary, processed products can be tolerated [5].

Currently, 26 potential proteins from tomato have been reported to provoke allergenic reactions, including different isoforms (http://www.allergome.org). Recently, two isoforms of the pathogenesis-related (PR) protein Sola l 4.01 and Sola l 4.02, homologous proteins to Bet v 1, the major birch pollen allergen from *Betula verrucosa*, have been identified [14]. Bet v 1.0101 (Acc. No. X15877, UniProt P15494) and the homologous proteins Sola l 4.01 (Acc. No. KF682291) and Sola l 4.02 (Acc. No. KF682292, UniProt K4CWC4) from tomato share 44.0 and 42.5% amino acid identity, respectively [14].

It has been shown that the allergenic potential of tomatoes is rather dependent on the cultivar and the developmental stages than environmental cultivation conditions [5]. Since numerous tomato cultivars are available, it might be possible that the concentration of Sola l 4 in some genotypes is sufficiently low, so that patients suffering from birch-pollen related tomato allergy can tolerate these.

Therefore, the aim of this study was to develop an enzyme-linked immunosorbent assay (ELISA) method to quantify Sola l 4 in various tomato cultivars with a specific polyclonal antibody. To analyze a wide range, varieties differing in size, shape and color were chosen. Furthermore, the influence of cultivation conditions (organic vs. conventional) as well as different processing methods (solar, oven and freeze-drying of tomato fruits) on the Sola l 4 content was investigated. It was hypothesized that the Sola l 4 content varies with the color of the mature fruits, the growing condition and the processing method. The results of this study could help to identify tomato fruits with a reduced allergen level to further improve food safety and life quality of birch pollen allergenic patients.

## Material and methods

### Chemicals

All chemicals were purchased from Sigma-Aldrich (Taufkirchen, Germany), Merck (Darmstadt, Germany) and Roth (Karlsruhe, Germany) unless otherwise noted.

### Plant material

Twenty-three different tomato varieties differing in color, size and shape (S1 Table) were provided by garden center Böck (Neufarn, Munich, Germany) and grown in the green house under equal temperature, light and water conditions. Fruits were harvested in July 2017 at full maturity (day post anthesis 40–45) of healthy plants without visible symptoms of pathogen infestation and stored at -20 °C until analysis. In the years 2015 and 2016, a local tomato cultivar SAAB and a commercial hybrid HF1 Perbruzzo were cultivated either conventionally or with two types of organic growing in the experimental field of CREA-OF (lat. 42° 53’ N, long. 13° 48’ E) in Central Italy Monsampolo del Tronto, Marche Region. Conventional cultivation soil was tilled and harrowed using Mater-Bi as artificially mulch (conv). Organic farming soil was coated with hairy vetch (*Vicia villosa* R.) and mulched either artificially with Mater-Bi (org) or naturally (norg) with mulch film out of lodged vetch. Fruits were harvested at full maturity in August 2015 and 2016.

### RNA isolation and cloning of Sola l 4.02

Commercially available tomatoes (cultivar Lyterno) were frozen in liquid nitrogen and homogenized to a fine powder using a Retsch mixer mill (Retsch MM400, Germany). Total RNA isolation and RNA precipitation were performed according to literature [15], except that the extraction buffer was prepared without spermidine. The concentration of the RNA preparation was determined with NanoDrop 1000 (Thermo Scientific, Germany) and the integrity was confirmed by agarose gel electrophoresis.

First strand cDNA synthesis was applied according to the manufacturer’s instructions (Promega, Germany). The open reading frame (ORF) sequence of the *Sola l 4*.*02* gene was amplified with PCR using gene-specific primers published from Wangorsch et al. [14]. PCR products were cloned into pGEM^®^-T Easy vector system according to manufacturer’s instruction (Promega, Germany). In order to obtain the expression vector the gene was amplified with two primers introducing a *Sph*I site (*Sph*I sola l 4 forward ACA TGC ATG CTT GGT GTA AAC ACC TTT ACT) and *Bgl*II site (*Bgl*II sola l 4 reverse CGGA AGA TCT AGC GTA GAC AGA AGG ATT) at its 5’end and 3’-end, respectively. The resulting PCR product was digested with *Sph*I and *Bgl*II and ligated into the predigested pQE70 vector (Quiagen, Hilden, Germany). After verification of the sequence by Eurofins (Ebersberg, Germany), pQE70-*Sola l 4*.*02* plasmid construct was transformed in *Escherichia coli* BL21 (DE3)pLysS (Novagen, Darmstadt, Germany).

### Heterologous expression and purification of Sola l 4.02 protein

Recombinant Sola l 4.02 was expressed in *Escherichia coli* (*E*. *coli*) BL21(DE3)pLysS as a fusion protein with a C-terminal His-tag. Cells were grown in 1 l LB medium supplemented with 100 μg/ml ampicillin and 34 μg/ml chloramphenicol at 37 °C to an optical density of 0.6. Gene expression was induced with 1 mM isopropyl β-D-1-thiogalactopyranoside (IPTG) and cultures were incubated for 20 h at 18 °C. Cells were harvested by centrifugation (10 min; 5292*g*; 4 °C) and cell pellets were stored at -80 °C.

Protein purification was performed via immobilized metal affinity chromatography using Profinity Immobilized Metal Ion Affinity Chromatography (IMAC) resin (Bio-Rad Laboratories, Germany). Cell pellets were suspended in 10 ml binding buffer (20 mM sodium phosphate pH 7.4; 0.5 M sodium chloride; 20 mM imidazole) and 0.5 mM PMSF, ultrasonicated and centrifuged for 30 min at 21191*g* at 4 °C. The supernatant containing soluble proteins was incubated for 2 h at 4 °C with the IMAC resin. After two washing steps with 10 ml of binding buffer each Sola l 4.02 protein was eluted with elution buffer (20 mM sodium phosphate pH 7.4; 0.5 M sodium chloride; 500 mM imidazole). The purity of the protein fractions was analyzed by SDS-PAGE. Five μg protein were separate in a 12% acrylamide stacking gel at 100 V for 2.5 h under non-reducing and reducing conditions with ß-mercaptoethanol. For protein staining Coomassie Brilliant Blue was used. PageRuler Prestained Protein Ladder (Thermo Scientific) was used as molecular weight marker. Elution fractions containing the respective protein were pooled and dialyzed against Phosphate-Buffered Saline (PBS) pH 7.4 at 4 °C for 20 h. Insoluble particles were removed by centrifugation and the protein solution was used as standard for indirect competitive ELISA.

### Production of polyclonal antibodies with specificity for Sola l 4.02

Specific polyclonal Sola l 4 antibody was produced by Davids Biotechnologie GmbH (Regensburg, Germany). Elution fractions of recombinant Sola l 4.02 protein purified from soluble fraction were pooled and used for immunization of rabbits according to a 63-day protocol. Antiserum was further purified via affinity chromatography using a column with Sola l 4 bound to the carrier matrix. Anti-Rabbit-Horseradish peroxidase (HRP) as secondary antibody was purchased from Carl Roth.

### Purification of Sola l 4.02 from insoluble fraction (inclusion bodies)

Sola l 4.02 was also purified from the insoluble fractions (inclusion bodies) to examine the ability of refolding of the protein and whether IgG recognition, analyzed by Western blot, was possible. The remaining pellet after cell lysis, ultra-sonication and centrifugation was used. The pellet was resolved in denaturation buffer (20 mM sodium phosphate pH 7.4; 0.5 M sodium chloride; 20 mM imidazole; 8 M urea) at 4 °C over night, centrifuged (1 h; 21191*g*; 4 °C) and refolded against refolding buffer (20 mM sodium phosphate pH 7.4; 0.5 M sodium chloride; 20 mM imidazole) at 4 °C over night via dialysis. After centrifuged (1 h; 21,200*g*; 4 °C) the supernatant was incubated with IMAC resin for 2 h at 4 °C. Protein purification was performed as described above for the soluble protein fraction. Purity of the protein fractions was evaluated by SDS-PAGE and Coomassie staining.

### Thermal treatment of rSola l 4.02

Five μg of recombinant Sola l 4.02 of purified pooled elution fractions from soluble fraction was incubated for 10, 20, 30, 60 and 90 min at 99 °C in a Thermoblock (Thermomixer comfort, Eppendorf) and immediately cooled on ice. Untreated protein solution was used as control. The integrity of the protein was further analyzed by SDS-PAGE. IgG binding was investigated via Western blot analysis using a specific polyclonal antibody against Sola l 4.02.

### Protein determination

The total protein concentration was determined in microtiter plates (Greiner 96 well plates, polypropylene, Sigma-Aldrich) using Roti^®^-Nanoquant following the manufacturer’s instructions (Carl Roth, Germany) with bovine serum albumin (BSA) as standard protein. Absorption at 450 nm and 590 nm was detected with the CLARIOstar plate reader (BMG Labtech, Germany).

### Drying of tomato fruits

Ripe fruits were cut into halves or quarters and further dried with three different methods upon constant dry weight. Oven drying was performed at 55 °C for 72 h in a conventional oven dryer (Thermo-Lab, Codogno, Italy). For solar drying only solar irradiance was used and performed in a miniaturized plant (TermoTend System-GTek, Carpi, Italy) for 7 to 10 days. Due to day-night-cycle, temperature varies between 25 °C and 45°C. Freeze-drying was performed for 96 h in an air-forced tunnel and lyophilized using a Dura-Stop tray dryer, combined with a Dura-Dry condenser module (FTS Systems, Stone Ridge, New York) from -35 °C to room temperature and samples were powdered before storage. The water loss was calculated from the difference between fresh and dried weight of the tomato fruit samples. All samples were stored at -20 °C until analysis. Dried fruits were compared to fresh, unprocessed tomatoes, which were only available in the year 2016.

### Tomato extracts

For the extraction of proteins from fresh tomatoes an established method [16] was applied. To reduce the intra- and inter-tomato variability of allergen distribution, eight frozen fruits of one variety were cut into halves or quarters, pooled and grind to a fine powder with a commercial blender (Personal Blender PB 250). For each variety protein extracts were prepared in triplicates. Tomato powder was supplemented with extraction buffer (10 mM KH_2_PO_4;_ 10 mM K_2_HPO_4_; 10 mM Na-DIECA; 2 mM EDTA; 2% (w/v) PVPP) containing 0.5 mM phenylmethylsulfonyl fluoride (PMSF) and protease inhibitor cocktail (Complete Protease Inhibitor Cocktail, Roche) 1:2 (w/v) and incubated at 4 °C for 4 h under shaking end over end. For dried plant material a ratio of 1:4 (w/v) was used to ensure proper mixing. Tomato extract were centrifuged for 15 min at 5292x*g* at 4 °C and dialyzed (3.4 kDa molecular weight cut-off, ZelluTrans, Carl Roth) against PBS pH 7.4. To remove any precipitates, a second centrifugation was performed for 10 min at 16100x*g* at 4 °C. Extracts were directly used for indirect competitive ELISA.

### Indirect competitive ELISA

The Sola l 4 content of tomato samples was determined by indirect competitive ELISA. Recombinant Sola l 4.02 from soluble *E*. *coli* fraction was used as competitor. Microtitre plates (immunoGrade^™^ Brand) were coated with 100 μl/well purified recombinant Sola l 4.02 protein (0.1 μg/ml) in coating buffer (PBS pH 7.4) and incubated at 4 °C overnight. After plates were washed three times with 300 μl washing buffer (0,05% (v/v) Tween 20 in PBS), free binding sites were blocked with 200 μl 2% BSA in PBS for 2 h at room temperature and washed as before. Dialyzed tomato extracts were diluted in washing buffer and 50 μl were pipetted to each well as “free” Sola l 4. Competition between immobilized and free allergen was performed by adding 50 μl of 2 μg/ml polyclonal Sola l 4-rabbit antibody and incubated for 4 h at 4 °C. Plates were washed four times and sequentially incubated with 100 μl of 1 μg/ml Anti-Rabbit-HRP (Carl Roth, Germany) for 1 h at room temperature. Following a final washing step, 100 μl of 1-Step Ultra 3,3’,5,5’-tetramethylbenzidine (TMB) ELISA solution (Thermo Scientific) were added and the color development was stopped with 100 μl 2 M sulfuric acid after 15 min. The absorption at 450 nm and 620 nm was measured with CLARIOstar plate reader (BMG Labtech, Germany). Standard curves and a negative control were applied on each microplate. The quantification of Sola l 4 allergen in tomato extracts was determined based on the standard curve with recombinant Sola l 4.02 protein. Fifty μl/well of serial dilutions (0.0001–25 μg/ml) of recombinant Sola l 4.02 was pipetted as “free” allergen and followed by the same procedure as the tomato samples. For data analysis the MARS software (BMG Labtech, Germany) was used. Sola l 4 content was expressed as μg Sola l 4 /g fresh weight respectively μg Sola l 4/ g dry weight. Dry matter was converted to fresh weight considering the loss of water in percent during drying process.

### Statistical analysis

For the analysis of the experimental data as well as for the box plots the statistical analysis software *R* (The R Foundation for Statistical Computing, R version i386 3.3.3) was used. Statistical significance levels between the variable groups were calculated using one-way analysis of variance (ANOVA). P values of ≤ 0.05 were considered as significant. For comparisons of mean values Tukey test was performed.

## Results

### Purification of recombinant Sola l 4.02 protein from soluble and insoluble (inclusion body) fraction

Recently, the two isoforms Sola l 4.01 and Sola l 4.02 have been identified as Bet v 1-related allergens in *Solanum lycopersicum* in tomato fruits from cultivar Verona [14]. Sola l 4.02 showed higher immunological activity in comparison to Sola l 4.01 and was therefore selected for the purpose of this study. The corresponding gene was isolated and cloned from tomato cultivar Lyterno showing complete sequence identity with the *Sola l 4*.*02* gene (Acc. No. KF682292; [14]).

**Fig 1:**
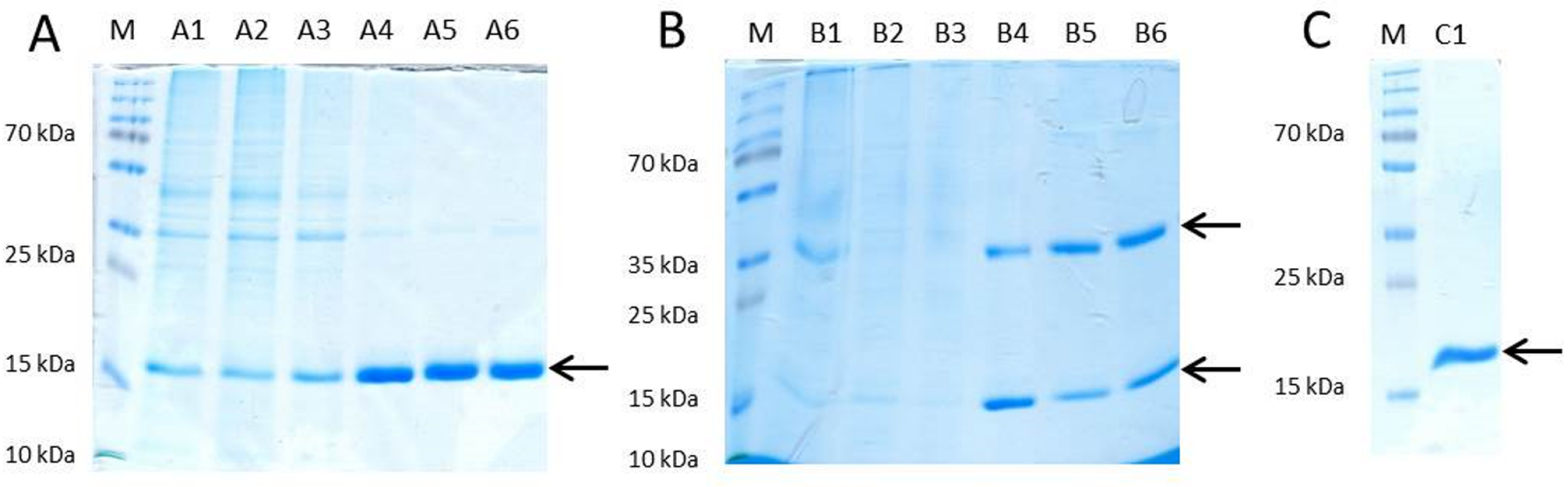
SDS-PAGE analysis of the recombinant Sola l 4.02 protein. (A) Purification from soluble protein fraction, SDS-PAGE under reducing condition with ß-mercaptoethanol (A1 crude extract; A2 flow through; A3 washing; A4 elution 1; A5 elution 2; A6 elution 3). (B) insoluble protein fractions, SDS-PAGE under non-reducing conditions (B1 denaturation; B2 refolding; B3 flow through; B4 elution 1; B5 elution 2; B6 elution 3). (C) insoluble protein fraction (C1 pooled elution 1–3). Under non-reducing conditions, (B) two distinct protein bands were visible in the elution fractions at 18 kDa and 36 kDa. Under reducing condition with ß-mercaptoethanol (A, C) only one band at approximately 18 kDa appeared. Five μg protein per lane were visualized by Coomassie Brilliant Blue G250. M: PageRuler Plus Prestained Protein Ladder.

Recombinant Sola l 4.02 was produced in *E*. *coli* BL21(DE3)pLysS and affinity purified from soluble and insoluble fraction, respectively. Whereas SDS-PAGE analysis of the Sola l 4.02 protein isolated from the soluble fraction showed only a band at the predicted molecular weight of 18 kDa (Fig 1A) Sola l 4.02 after denaturation and refolding from inclusion bodies displayed a second band with a molecular weight of approximately 36 kDa (Fig 1B). SDS-PAGE under reducing conditions with ß-mercaptoethanol showed only one specific band at 18 kDa (Fig 1C).

Specific polyclonal antibodies against Sola l 4 were produced via immunization of rabbit with purified recombinant protein from the soluble fraction. Western Blot analysis showed that the antibody recognized both, the soluble Sola l 4.02 as well as the refolded protein from inclusion bodies (S1 Fig). Furthermore, Western Blot analysis confirmed that the polyclonal antibody specifically recognizes native Sola l 4 allergen extracted from tomato fruits (S2 Fig).

### Thermal treatment of recombinant Sola l 4.02 protein

**Fig 2:**
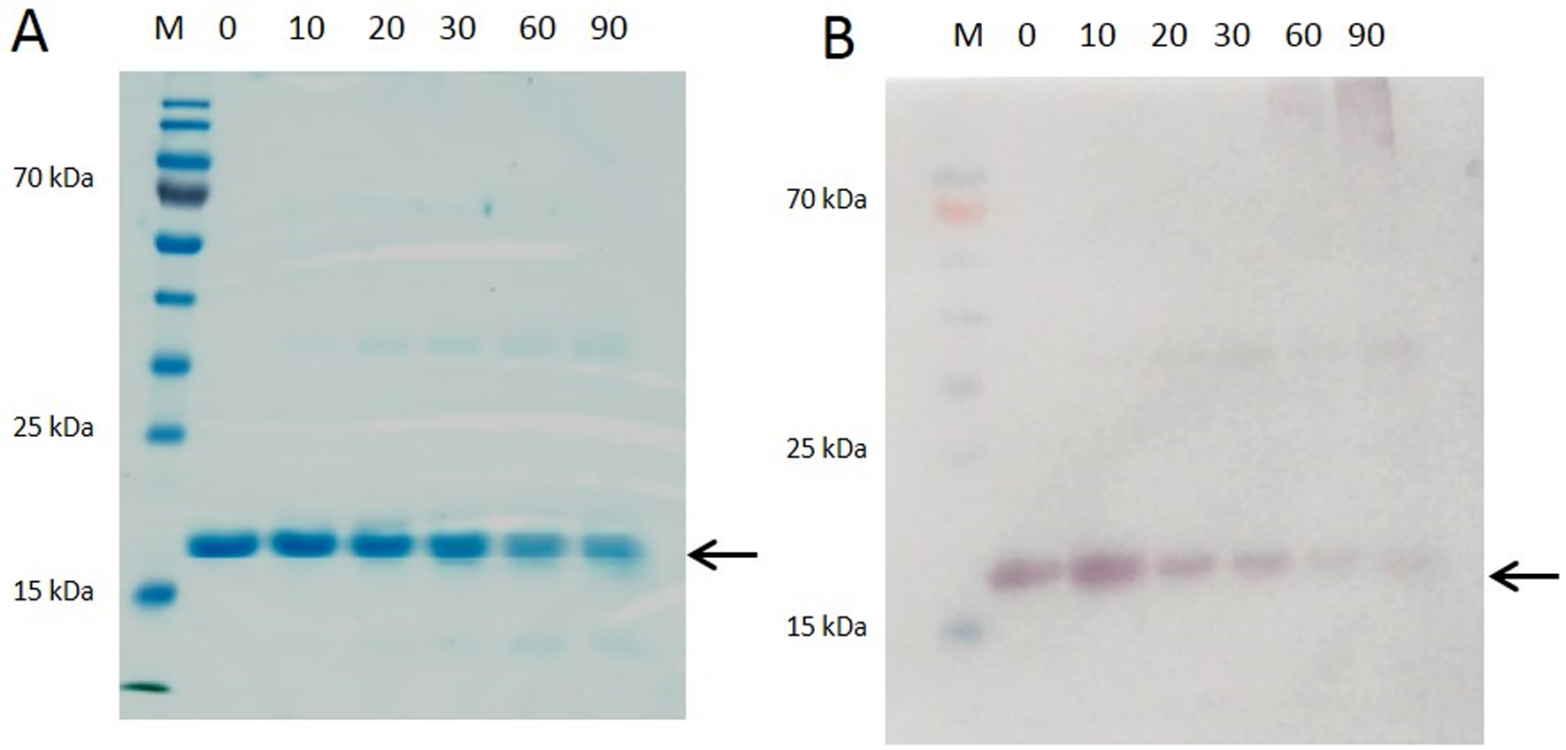
Heat stability of soluble recombinant Sola l 4.02. (A) SDS-PAGE and (B) Western-Blot analysis of purified pooled elution fractions of the recombinant Sola l 4.02 protein heated for 10, 20, 30, 60 and 90 min at 99 °C. Untreated protein served as control (0). SDS-PAGE was performed under reducing conditions. Coomassie Brilliant Blue G250 was used for protein staining. For Western blot analysis specific polyclonal Sola l 4-antibody was used. M: PageRuler Prestained Protein Ladder.

Pooled elution fractions of purified recombinant Sola l 4.02 from the soluble protein fraction were thermally treated and analyzed by SDS-PAGE (Fig 2A) and Western blot (Fig 2B) to investigate the effect of heat on integrity and IgG recognition. After 10 to 30 min at 99 °C the rSola l 4.02 protein was still detectable showing a clear band at 18 kDa in Coomassie stained SDS-PAGE gel (Fig 2A). Prolonged heating of 60 min or even of 90 min resulted in diffuse protein bands. Moreover, IgG-binding activity decreased considerably already after 10 min of thermal treatment of the Sola l 4.02 protein and was barely visible after 90 min at 99 °C (Fig 2B).

### Validation of the ELISA and extraction method

An indirect competitive ELISA was developed using recombinant Sola l 4.02 as solid phase-bound antigen and as standard protein to determine the Sola l 4 content in various fresh and dried tomato samples. A polyclonal rabbit antibody directed to Sola l 4 was used to detect the Bet v 1-related allergen in tomato extracts. The ELISA showed a typical standard curve ranging was from 0.01 to 10.0 μg/ml (S3 Fig). To reduce the intra- and inter-tomato variability of allergen distribution, fine powder of eight frozen fruits of one variety was pooled and further used for protein extraction. Protein extracts were prepared in triplicates and extracts were diluted 2- and 4-fold for ELISA measurement. Finally the Sola l 4 content of one variety was calculated for the three extraction replicates from two dilutions and three technical replicates on the microtiter plate. The Sola l 4 content measured with ELISA was comparable in all three protein extracts (S3 Fig).

### Influence of tomato variety on Sola l 4 content

**Fig 3:**
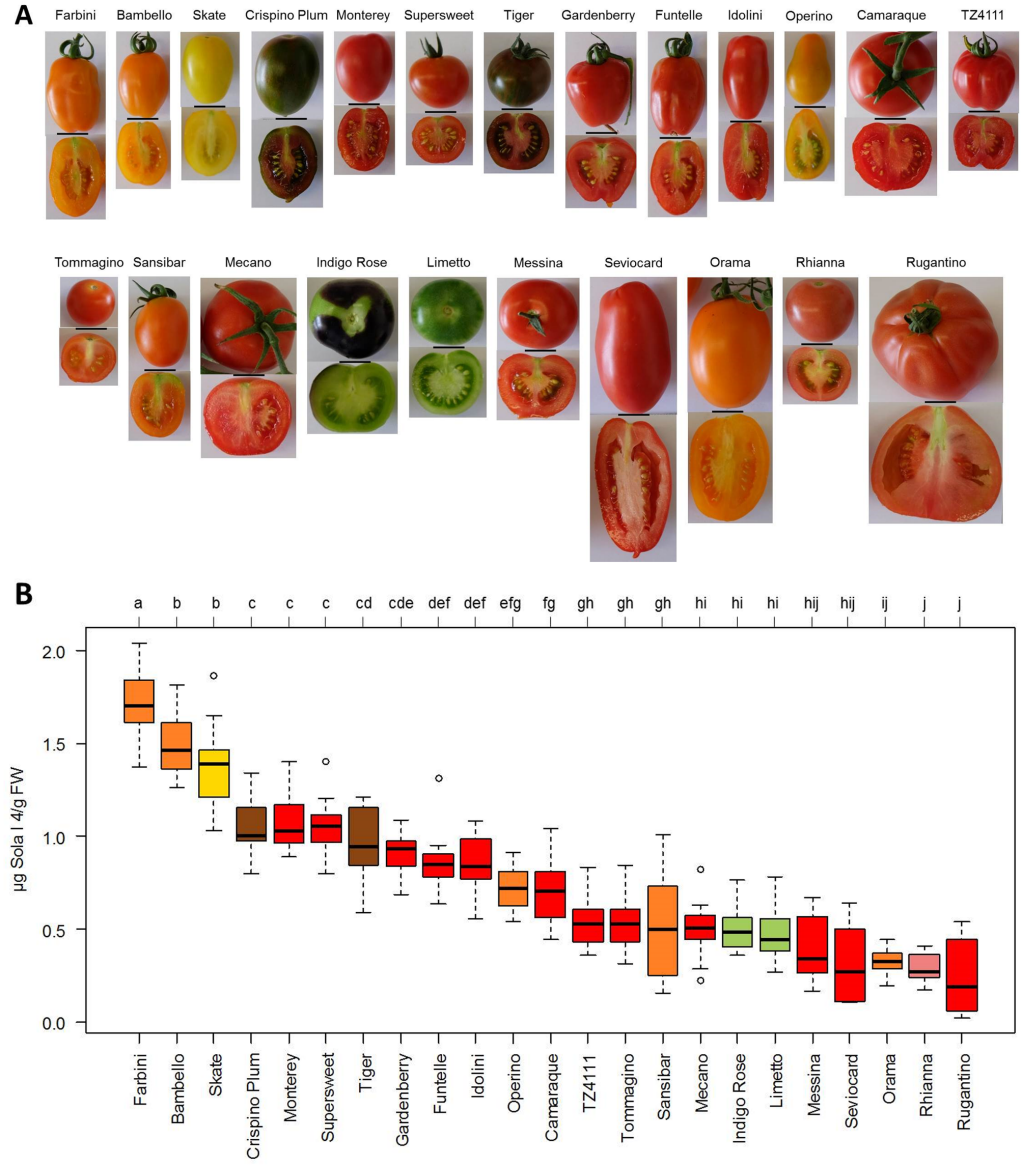
Sola l 4 content in different tomato cultivars. (A) Diversity of tomato cultivars (bar = 2 cm) and (B) corresponding Sola l 4 content in μg/g fresh weight (FW) determined with indirect competitive ELISA. The color of the box plots corresponds to the color of the ripe tomato fruit. Significant differences for each cultivar were calculated at a significance level of 5%.

Twenty-three different colored tomato varieties of varying sizes and shapes were investigated to analyze the genetic (cultivar-to-cultivar) factor on the expression of Sola l 4 at a translational level in the fruit (Fig 3A). Total protein levels of 113.5 to 584 μg soluble protein /g fresh weight (FW) could be extracted from fresh tomatoes (S1 Table). Sola l 4 levels ranged from 0.24 to 1.71 μg/g FW (Fig 3B). The colors of the box plots represent the respective fruit color. The significantly lowest level of Sola l 4 was found in the cultivars Rugantino and Rhianna with 0.24 and 0.29 μg Sola l 4/g FW, respectively whereas the significantly highest concentration was determined in cultivars Farbini and Bambello with 1.71 and 1.5 μg Sola l 4/g FW, respectively. Ten significance groups (letters a-j) were calculated according to the Tukey Test with 5% of significance level. The percentage of Sola l 4 referred to the total soluble protein amount varied between 0.094% for cultivar Rugantino and 0.658% for cultivar Supersweet (S1 Table).

### Effect of cultivation and processing methods on Sola l 4 content in dried tomatoes

The influence of cultivation conditions, seasonal effects and processing techniques on the Sola l 4 allergen content in dried tomatoes was studied by ELISA. The two cultivars SAAB and Perbruzzo are genotypes well adapted for the growing conditions in Central East Italy. SAAB is very suitable for growing in organic crop management whereas Perbruzzo, a similar type is more adapted for commercial purposes. Tomatoes were grown in Italy in the years 2015 and 2016 either conventionally or organically, with further classification into conventionally grown with artificial mulch (conv), organically grown with artificial mulch (org) and organically grown with natural mulch (norg). After harvest, ripe fruits were dried in the oven (oven), in the sun (solar) or via freeze-drying (freeze). Water loss in percent was calculated from the difference of fresh and dry weight and was further included for conversion of allergen content of dry matter to fresh matter.

**Fig 4:**
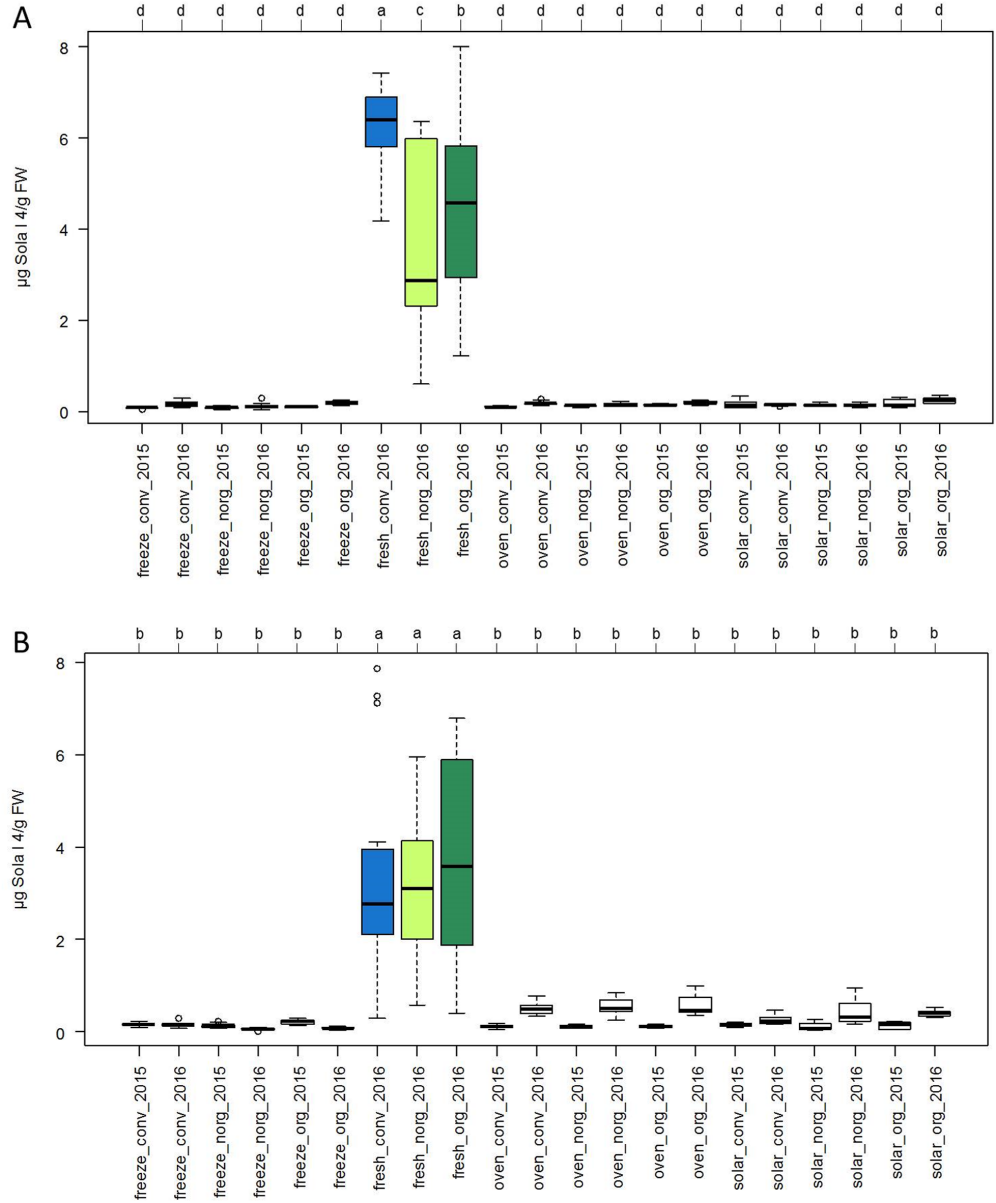
Sola l 4 content in dried and fresh tomatoes. (A) Allergen content in μg Sola l 4/g fresh weight (FW) of tomato cultivars SAAB and (B) Perbruzzo determined with indirect competitive ELISA. Plants were grown in Italy in 2015 and 2016 conventionally with artificial mulch (conv), organically with artificial mulch (org) and organically with natural mulch (norg). Tomato fruits were dried via freeze-drying (freeze), in the oven (oven) and in the sun (solar). Allergen content of dried tomato samples was referred to μg Sola l 4/g FW and compared with fresh tomatoes (fresh). Significant differences for each group were calculated at a significance level of 5%.

Dried tomato products of both genotypes contained significantly lower levels of Sola l 4 than the fresh fruits (Fig 4), when referring the allergen content of the samples to the corresponding fresh weight, regardless of the cultivation technique. Compared to dried fruits, fresh SAAB and Perbruzzo tomatoes of 2016 contained between 3.66 to 6.25 μg and 3.19 to 3.74 Sola l 4/g FW, respectively.

**Fig 5:**
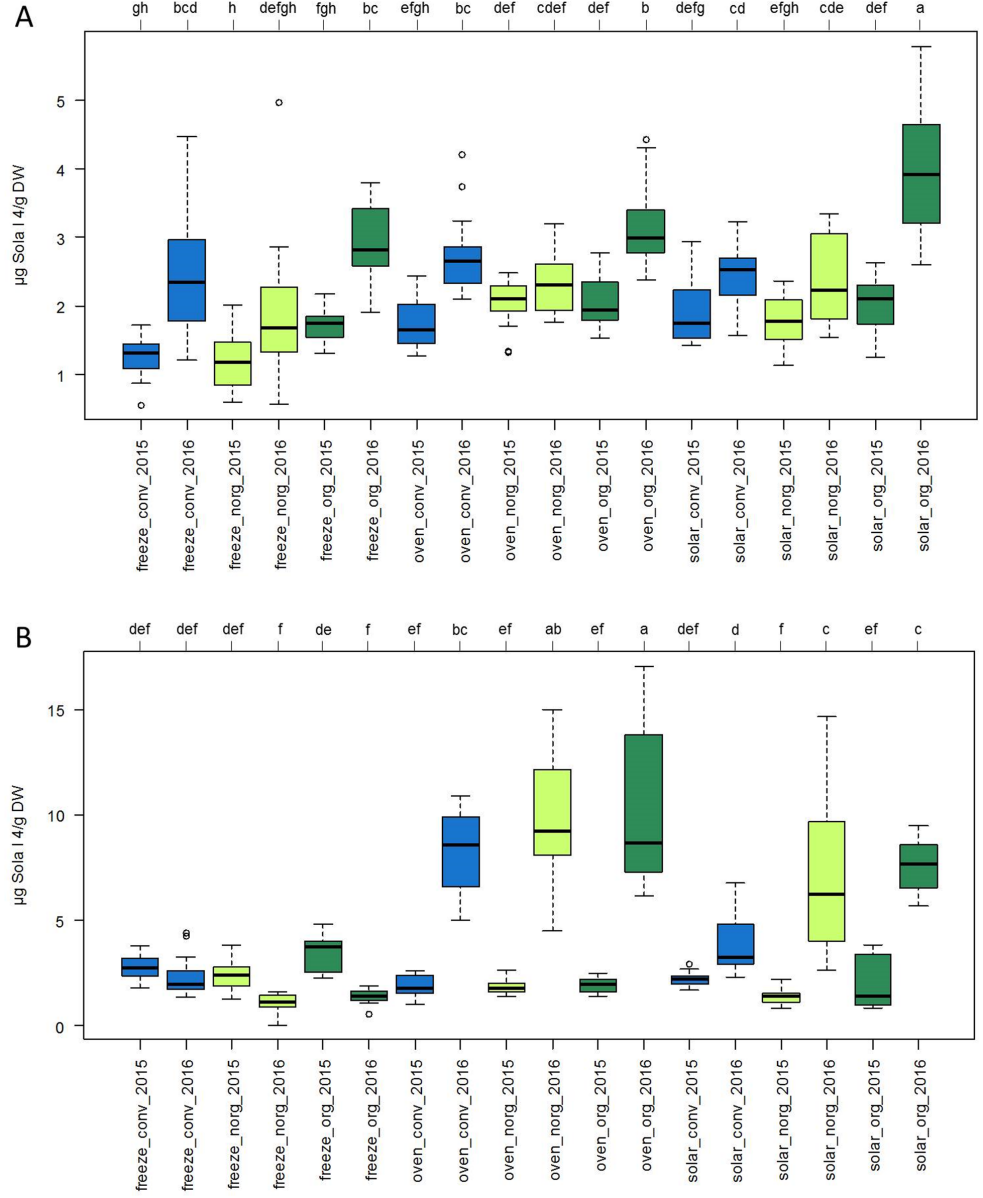
Effect of cultivation and drying method on Sola l 4 content in dried tomatoes. (A) Allergen content in μg Sola l 4/g dry weight (DW) of tomato cultivars SAAB and (B) Perbruzzo determined with indirect competitive ELISA. Plants were grown in Italy in 2015 and 2016 conventionally with artificial mulch (conv), organically with artificial mulch (org) and organically with natural mulch (norg). Tomato fruits were dried via freeze-drying (freeze), in the oven (oven) and in the sun (solar). Significant differences for each group were calculated at a significance level of 5%.

In the dried products of the SAAB genotype the allergen content ranged from 1.24 μg Sola l 4/g dry weight (DW) for freeze-dried tomatoes grown organically with natural mulch in 2015 up to 3.93 μg Sola l 4/g DW for solar dried fruits grown organically with artificial mulch in 2016 (Fig 5A). This corresponded to 0.07 and 0.23 μg Sola l 4/g FW, respectively (Fig 4A). When comparing the two consecutive years, all dried samples of the SAAB genotype from 2016 showed higher allergen content than samples from 2015 (Fig 5A) with significant effects for some samples. No significant differences were observed for the influence of the cultivation method when comparing dried SAAB tomato samples from one year and fruits were dried with the same method. Furthermore, there were no significant differences between the three drying methods when comparing dried SAAB samples from one year and plants were grown under the same conditions.

The allergen content of dried Perbruzzo samples (Fig 5B) ranged from 1.04 μg Sola l 4/g DW for freeze-dried tomatoes grown organically in 2016 with natural mulch up to 10.28 μg Sola l 4/g DW for oven dried fruits grown organically with artificial mulch in 2016. This corresponded to 0.05 and 0.58 μg Sola l 4/g FW (Fig 4B). Compared to fresh fruits dried tomatoes showed a significantly lower allergen content (Fig 4). When comparing the two consecutive years, oven and solar dried samples from cltivar Perbruzzo showed higher allergen content in 2016 than samples from 2015 (Fig 5B) with significant effects. Strikingly, freeze-dried tomatoes exhibited no significant differences between the two years. The influence of the cultivation method showed no significant effect on the allergen content of Perbruzzo products when comparing dried tomatoes from one year and fruits were dried with the same method.

## Discussion

In Northern Europe, individuals allergic to birch pollen often show cross-reactivity to allergens from *Rosaceae* fruits or other vegetables and nuts [17]. IgE antibodies directed to Bet v 1 induced in a primary sensitization reaction to birch pollen can also react with Bet v 1-related proteins from various plant origin [18]. Here, we have analyzed the Bet v 1-like Sola l 4.02 protein of the PR-10 family whose gene was identified in the *S*. *lycopersicum* genome only recently [14]. We studied the effects of the genotype, cultivation, climate and processing methods on the level of Sola l 4.

### Biochemical and immunological properties of recombinant Sola l 4.02

The protein Sola l 4.02 (K4CWC4) shares 42.5% amino acid similarity with Bet v 1.0101 (P15494). SDS-PAGE analysis of the recombinant protein under nonreducing conditions showed that Sola l 4.02 exists in a monomeric and a dimeric form, with a molecular weight of 18 kDa and 36 kDa, respectively (Fig 1). Two cysteine residues at position C113 and C115 might be able to form disulfide bonds. SDS-PAGE under reducing conditions with ß-mercaptoethanol resulted in only one band at 18 kDa, probably due to the cleavage of the disulfide bond. Dimerization did not affect the structure of the protein epitopes as the binding of IgG antibodies and immunological reactivity of the dimers maintained (Fig 1). Dimerization or even oligomerization of recombinant allergens and naturally-occurring allergens was observed previously [19–21]. Similarly, Bet v 1 has been reported to exist as a dimer [22–25]. Although Bet v 1.0101 does not comprise a cysteine residue in its amino acid sequence and the mechanism of dimerization has not been fully elucidated, dimer formation can be induced by mutation of position 5 to a cysteine residue [24].

Besides of the property of the polyclonal antibody to recognize recombinant Sola l 4 purified from soluble fraction, the purification of Sola l 4 from insoluble inclusion body fraction showed that antibody binding to the refolded protein occurs. After denaturation with urea and refolding of Sola l 4, essential epitopes for antigen-antibody reaction must be present (S1 Fig). An important observation is, that the antibody recognizes specifically the allergen extracted from tomato fruits (S2 Fig). Purification of proteins from cell pellet under denaturating conditions and refolding is a common method, when the amount of soluble protein is too low. High purity and application in enzyme allergosorbent test, Western blots and basophil histamine release were described for recombinant Pyr c 1, showing similar allergenic activity to the natural allergen from pear [26]. Furthermore, specific IgE from pear-allergenic patient sera recognized the recombinant protein, purified from inclusion body fraction.

PR-10 (Bet v 1 related) proteins from *Rosaceae* family are unstable to heat and sensitive to proteases. Therefore, allergic symptoms are restricted to the upper intestinal tract (mouth) since Bet v 1-related proteins are digested by proteases in the lower intestinal tract [27]. Similarly, heat inactivation of rSola l 4.02 purified from soluble protein fraction was demonstrated (Fig 2). Thermal treatment of the recombinant allergen changed the protein structure in such a way, that recognition of the allergen epitopes by anti-Sola l 4 was remarkably reduced (Fig 2). The same phenomenon has been shown for rPru av 1 from cherry [27] and the apple allergen Mal d 1 [28].

### Allergenic potential of tomatoes is cultivar dependent

Tomato allergy is often accompanied with pollen allergies [29]. Depending on the regional distribution of pollen allergens, tomato allergic patients can be sensitized towards several tomato allergens from different protein families [5]. The best-known groups are allergens homologous to Bet v 1, profilins, and lipid transfer proteins (LTP). Tomato allergy is more common in Southern Europe where allergic reactions are caused by the major allergens Sola l 6 and Sola l 7, proteins belonging to non-specific LTP [3]. These allergens are heat stable and provoke severe symptoms. However, in Northern Europe Bet v 1 related Sola l 4 allergy is prevalent. Sola l 4 was recognized in 76% of birch/tomato allergic patients highlighting Sola l 4 as major allergen in tomato fruits [14].

Thus, an indirect competitive ELISA was established using a polyclonal antibody directed to Sola l 4.02 and differently colored tomato genotypes were analyzed as fruit color has been recently correlated with allergen content [30]. Among 23 different varieties, the allergen content varied between 0.24 and 1.71 μg Sola l 4/g FW, independent of the total soluble protein amount respectively the percentage of Sola l 4 allergen and the color. The high variation in allergen content supports recent results, which showed that patients exhibited different antibody-binding profiles because of varying allergenic activities of tomato cultivars verified with skin prick tests and basophil activation test [31]. Besides, it seems that Sola l 4 does not function in carotenoid biosynthesis, the major group of colorants in tomato.

Fresh tomato fruits from cultivars SAAB and Perbruzzo from Italy show generally higher Sola l 4 allergen content compared to the collection of varieties from garden center Böck (Germany). Different locations and climatic conditions are an important parameter affecting [32] the allergen level, previously shown for Mal d 1 content in apples. Besides this, tomatoes from Germany were cultivated in the greenhouse.

Allergenicity of fruits is cultivar dependent as evaluated for the major apple allergen Mal d 1 [32,33]. Allergen level of the Bet v 1-homologous Mal d 1 in apple varied between 3.8 and 72.5 μg/g pulp [33] or between 2.3 and 20.1 μg/g FW [32]. Thus, the content of Bet v 1-homologous proteins in apples is higher than the corresponding protein in tomatoes (Fig 3). Apple allergies affect up to 2% of the population in Europe and Northern America. The prevalence of tomato allergies caused by PR-10 related allergens, however, is rare. The lower Sola l 4 allergen level in tomato compared to apple fruits might be a reason for that. Especially in the Mediterranean area tomato allergy is more relevant with severe symptoms provoked by allergens from LTPs and profilins.

It has to be taken into account that, in addition to Sola l 4.01 and Sola l 4.02, additional isoforms might be expressed in tomato fruits playing a role for PR-10 allergenic patients. The severity of an allergic reaction to fruit is related to the individual sensitivity of the patient and moreover depended on the cultivar. Identification of specific IgE-antibodies in patient sera and skin prick test with different varieties reveal in most of the cases a wide range from low to high allergenic reactivity. Thus, the results of the ELISA have to be confirmed by further immune tests but can be helpful to improve the quality of tomato cultivars.

### Drying processing of tomato fruits has major effect on allergen content

During food processing the allergenic properties of food allergens can be altered by various parameters. Washing or peeling of the food material, breaking up through grinding or cutting, thermal treatment, fermentation processes or even purification steps in the manufacturing procedure may have an effect on the allergenic properties of food allergens [34]. Changes in epitope protein structure can be the factor for both, decreasing or increasing allergenic activity.

Non-specific LTPs are a major elicitor of tomato allergies. Both, in fresh fruits as well as in industrial products LTP are contained in crucial amounts, triggering severe allergic symptoms [35]. Due to the high resistance to proteases and heat, these proteins maintain their immunological activity [3]. In contrast, Bet v 1 related proteins are heat-labile and patients allergic to PR-10 proteins might tolerate processed food or food products. The loss of allergenicity due to thermal processing was investigated for several Bet v 1-related allergens, such as Mal d 1 [28] and Pru av 1 [27]. Furthermore, we showed that recombinant Sola 1 4.02 is also heat sensitive (Fig 2).

Since dried tomatoes are a common product in food industry, the Sola l 4 amount was determined in a number of differently dried fruits. Due to thermal treatment, the level of the Sola l 4 allergen decreased significantly (Fig 4). Considering the loss of water during the drying process, dried tomatoes contain considerably lower Sola l 4 amount than fresh tomatoes. Both, the experiment with the recombinant protein and with tomato extracts from dried fruits affirm the heat-sensitivity of this PR-10 protein. Although freeze-drying is a gentle drying method known to preserve the protein structure, freeze-dried tomato samples contained the same low allergen content as oven and solar dried fruits. Due to the loss of water during drying, the protein structure of the soluble Sola l 4 protein becomes altered and is not recognized any more by the antibody. In addition to that, the protein might be degraded and therefore the antibody is unable to recognize the protein fragments. No significant changes in Sola l 4 levels between freeze-, solar-, and oven drying were observed for cultivar Perbruzzo in 2015 (Fig 5). In contrast, differences were detected in 2016. Removal of water by oven and solar-drying seemed to be less effective in 2016 promoting protein solubility and allergen stability. For cultivar SAAB the differences were insignificant between freeze-, solar-, and oven drying in both years 2015 and 2016.

According to the meteorological data (Table 1) from the growing region of tomato cultivars SAAB and Perbruzzo in Monsampolo del Tronto, the rainfall was significantly higher in 2016 than in the previous year. From May to August 2015 167.6 liter per square meter were measured, compared to 267.2 liter per square meter during the same season in the following year. The average daily temperatures from May to August were slightly lower in 2016 with 21.6 °C compared to 2015 with 22.9 °C, which is in accordance with higher rainfall in 2016. Sola l 4 allergen levels of dried tomato fruits were higher in 2016 than in 2015 for the majority of analyzed samples. Due to strong rain and high humidity, the pathogen infestation is increased and might lead to upregulation of PR-10 genes. Thus, varying weather conditions including average temperature, precipitation and humidity seem to have a more important effect on the allergen content than conventional or organic growing including the fact that pathogen growing is promoted under specific climatic conditions leading to induction of PR-10 genes. We conclude that growing conditions and seasonal effects such as low humidity and high temperature, which reduce the propagation of pathogens, would also reduce Sola l 4 content.

**Table 1:** Meteorological data at the growing location Monsampolo del Tronto, Italy for the years 2015 and 2016.

month	mean temperature [°C]	rainfall [mm]
May 2015	18.2	56.2
June 2015	21.7	75.0
July 2015	26.9	0.4
August 2015	24.8	36.0
May 2016	16.4	69.8
June 2016	21.3	98.8
July 2016	25.1	72.2
August 2016	23.5	26.4

## Conclusion

In summary, the level of Bet v 1-related allergen in tomato fruits varied significantly between cultivars. Furthermore, the heat sensitivity of the PR-10 protein Sola l 4 was confirmed for the recombinant protein as well as for tomato samples, when fruits were exposed to heat during the drying process. Sola l 4.02 may be a marker for breeding hypoallergenic tomato varieties.

## Supporting information

S1 Fig Recombinant Sola l 4.02 protein purified from inclusion body fraction. (A) SDS-PAGE and (B) Western-Blot analysis of pooled elution fractions of the recombinant Sola l 4.02 protein purified from insoluble fraction (IB). SDS-PAGE was performed under reducing conditions. Coomassie Brilliant Blue G250 was used for protein staining. For Western blot analysis specific polyclonal Sola l 4-antibody was used. M: PageRuler Prestained Protein Ladder.
(PDF)

S2 Fig Protein pattern of tomato extracts. (A) SDS-PAGE and (B) Western-Blot analysis of native tomato protein extracts exemplarily shown for different commercially available tomato cultivars. SDS-PAGE was performed under reducing conditions. Coomassie Brilliant Blue G250 was used for protein staining. For Western blot analysis specific polyclonal Sola l 4-antibody was used. The 18 kDa band, corresponding to the native Sola l 4, is marked with an arrow. M: PageRuler Prestained Protein Ladder.
(PDF)

S3 Fig Indirect competitive ELISA and protein extraction. (A) Standard curve of indirect competitive ELISA to quantify Sola l 4 in tomato and (B) reproducibility of the protein extraction method exemplarily shown for cultivars Farbini, Gardenberry and Orama.
(PDF)

S1 Table Tomato cultivars. Sola l 4 content (mean values) in μg/g fresh weight (FW), total soluble protein in μg/g FW and percentage of Sola l 4/total soluble protein of different tomatoes. Plants were grown at garden center Böck (Neufahrn, Munich).
(PDF)
