# Supplementary material for: Effect of tomato variety, cultivation, climate and processing on Sola l 4, an allergen from Solanum lycopersicum
Source: PLoS One. 2018 Jun 14;13(6):e0197971. doi: 10.1371/journal.pone.0197971 (PMC6002116; doi:10.1371/journal.pone.0197971)
Supplement: S1 Fig — (A) SDS-PAGE and (B) Western-Blot analysis of pooled elution fractions of the recombinant Sola l 4.02 protein purified from insoluble fraction (IB). SDS-PAGE was performed under reducing conditions. Coomassie Brilliant Blue G250 was used for protein staining. For Western blot analysis specific polyclonal Sola l 4-antibody was used. M: PageRuler Prestained Protein Ladder. (PDF) [file pone.0197971.s001.pdf]

Supporting information

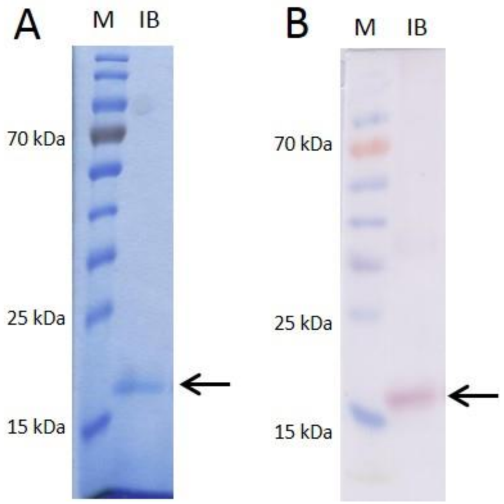

S1 Fig. Recombinant Sola l 4.02 protein purified from inclusion body fraction. (A) SDS-PAGE and (B) Western-Blot analysis of pooled elution fractions of the recombinant Sola l 4.02 protein purified from insoluble fraction (IB). SDS-PAGE was performed under reducing conditions. Coomassie Brilliant Blue G250 was used for protein staining. For Western blot analysis specific polyclonal Sola l 4-antibody was used. M: PageRuler Prestained Protein Ladder.
