# Supplementary material for: Effect of tomato variety, cultivation, climate and processing on Sola l 4, an allergen from Solanum lycopersicum
Source: PLoS One. 2018 Jun 14;13(6):e0197971. doi: 10.1371/journal.pone.0197971 (PMC6002116; doi:10.1371/journal.pone.0197971)
Supplement: S2 Fig — (A) SDS-PAGE and (B) Western-Blot analysis of native tomato protein extracts exemplarily shown for different commercially available tomato cultivars. SDS-PAGE was performed under reducing conditions. Coomassie Brilliant Blue G250 was used for protein staining. For Western blot analysis specific polyclonal Sola l 4-antibody was used. The 18 kDa band, corresponding to the native Sola l 4, is marked with an arrow. M: PageRuler Prestained Protein Ladder. (PDF) [file pone.0197971.s002.pdf]

Supporting information

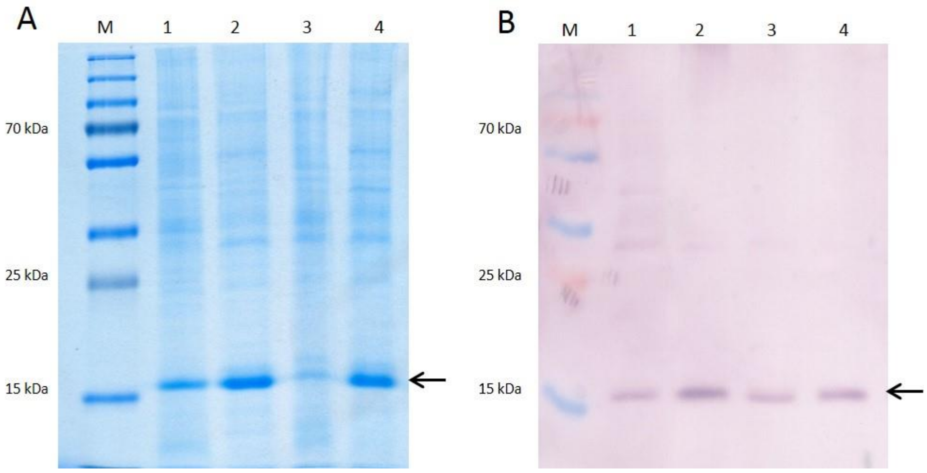

S2 Fig. Protein pattern of tomato extracts. (A) SDS-PAGE and (B) Western-Blot analysis of native tomato protein extracts exemplarily shown for different commercially available tomato cultivars. SDS-PAGE was performed under reducing conditions. Coomassie Brilliant Blue G250 was used for protein staining. For Western blot analysis specific polyclonal Sola l 4-antibody was used. The 18 kDa band, corresponding to the native Sola l 4, is marked with an arrow. M: PageRuler Prestained Protein Ladder.
