# Supplementary material for: Effect of tomato variety, cultivation, climate and processing on Sola l 4, an allergen from Solanum lycopersicum
Source: PLoS One. 2018 Jun 14;13(6):e0197971. doi: 10.1371/journal.pone.0197971 (PMC6002116; doi:10.1371/journal.pone.0197971)
Supplement: S3 Fig — (A) Standard curve of indirect competitive ELISA to quantify Sola l 4 in tomato and (B) reproducibility of the protein extraction method exemplarily shown for cultivars Farbini, Gardenberry and Orama. (PDF) [file pone.0197971.s003.pdf]

Supporting information

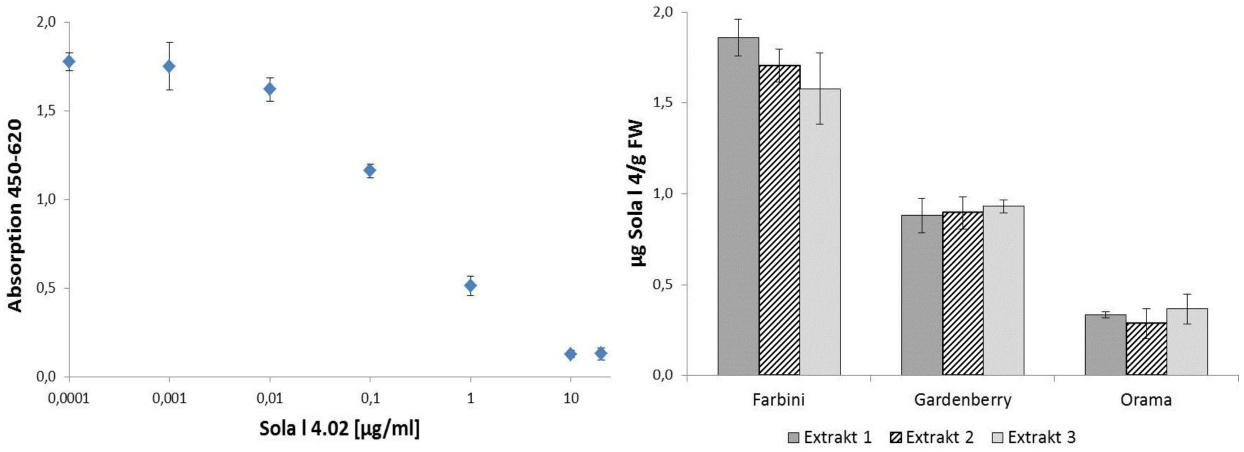

S3 Fig. Indirect competitive ELISA and protein extraction. (A) Standard curve of indirect competitive ELISA to quantify Sola l 4 in tomato and (B) reproducibility of the protein extraction method exemplarily shown for cultivars Farbini, Gardenberry and Orama.
