# Supplementary material for: Effect of tomato variety, cultivation, climate and processing on Sola l 4, an allergen from Solanum lycopersicum
Source: PLoS One. 2018 Jun 14;13(6):e0197971. doi: 10.1371/journal.pone.0197971 (PMC6002116; doi:10.1371/journal.pone.0197971)
Supplement: S1 Table — Sola l 4 content (mean values) in μg/g fresh weight (FW), total soluble protein in μg/g FW and percentage of Sola l 4/total soluble protein of different tomatoes. Plants were grown at garden center Böck (Neufahrn, Munich). (PDF) [file pone.0197971.s004.pdf]

Supporting information

S1 Table. Tomato cultivars. Sola l 4 content (mean values) in µg/g fresh weight (FW), total soluble protein in µg/g FW and percentage of Sola l 4/total soluble protein of different tomatoes. Plants were grown at garden center Böck (Neufahrn, Munich).

cultivar	color	Sola l 4 [µg/g FW]	total soluble protein [µg/g FW]	% Sola l 4/total soluble protein
Indigo Rose	green/black peel	0.493 ± 0.11	291.1 ± 19.2	0.169
Limetto	green	0.489 ± 0.17	144.1 ± 10.2	0.339
Skate	yellow	1.363 ± 0.20	229.0 ± 14.0	0.595
Bambello	orange	1.497 ± 0.15	584.0 ± 19.9	0.256
Farbini	orange	1.714 ± 0.19	380.9 ± 4.7	0.450
72-187 Operino	orange	0.718 ± 0.11	423.3 ± 21.1	0.170
Orama	orange	0.328 ± 0.06	292.1 ± 9.0	0.112
Sansibar	orange	0.534 ± 0.30	298.3 ± 3.7	0.179
Rhianna	rose	0.292 ± 0.08	113.5 ± 13.3	0.257
Camaraque	red	0.698 ± 0.16	474.3 ± 12.2	0.147
Funtelle	red	0.855 ± 0.15	291.8 ± 9.3	0.293
Gardenberry	red	0.902 ± 0.11	318.3 ± 6.0	0.283
Idolini	red	0.854 ± 0.15	411.6 ± 19.8	0.207
Mecano	red	0.501 ± 0.13	313.4 ± 14.1	0.160
Messina	red	0.384 ± 0.16	144.0 ± 3.1	0.267
Monterey	red	1.065 ± 0.14	394.4 ± 21.0	0.270
Rugantino	red	0.244 ± 0.19	259.7 ± 10.0	0.094
Seviocard	red	0.337 ± 0.22	261.9 ± 11.1	0.129
Supersweet	red	1.050 ± 0.14	159.5 ± 7.1	0.658
Tommagino	red	0.535 ± 0.15	140.2 ± 5.5	0.382
TZ 4111	red	0.551 ± 0.13	217.8 ± 3.2	0.253
Crispino Plum	brown	1.067 ± 0.14	480.6 ± 15.8	0.222
Tiger	brown/green	0.967 ± 0.19	295.7 ± 20.5	0.327
